# THE PROTECTIVE LIFESPAN BENEFITS OF SOCIAL SUPPORT BUFFERS CHILDHOOD ADVERSITY

**DOI:** 10.1093/geroni/igad104.3652

**Published:** 2023-12-21

**Authors:** Meredith Willard, Ryan Best, Nicholas Turiano

**Affiliations:** West Virginia University, Morgantown, West Virginia, United States; West Virginia University, Morgantown, West Virginia, United States; West Virginia University, Morgantown, West Virginia, United States

## Abstract

Childhood adversity has negative effects across the lifespan including increased mortality risk. The support we receive from others, social support, has shown to be a protective factor against ACEs. However, little research has investigated the amplifying effects of poor social support, also known as social strain. Individuals with high levels of social strain and low levels of adversity would have the lowest mortality risk, it was hypothesized. Early life adversity was computed using 16 retrospective items assessing emotional and physical abuse, socioeconomic disadvantage, familial instability, and early-life poor health. Social support was computed using four-family, four-friend, and six-spouse items assessing reliability and emotion availability. Social strain was computed using four-family, four-friend, and six-spouse items assessing annoyance and disappointment. Cox proportional hazards models tested whether adversity, social support, and social strain were associated with the 20+year hazard of dying. Adjusting for age, gender, race, education, and marital status, higher ACEs (HR = 1.06) lower social support (HR = 1.07) and greater social strain (HR = 1.06) were all significantly associated with an increased hazard of dying (all ps < .05). A significant ACE x Social Support interaction emerged (HR = 0.95) suggesting the association between ACEs and hazard of dying was strongly positive at low levels of social support, but that the relationship was negative at high levels of support. Thus, high levels of social support buffer the negative effects ACEs have on mortality risk. This provides robust evidence that interventions increasing social support could reduce the harmful effects ACEs.

